# Epilepsy Treated by Lactate of Zinc

**Published:** 1867-07

**Authors:** C. F. Hart

**Affiliations:** Chicago


### EPILEPSY TREATED BY LACTATE OF ZINC.

By C. F. Hart, M.D., Chicago.

In looking over some notes on my early reading, I find a memorandum of Dr. Herpen's remedy for erysipelas, by which he has made some complete cures. The remedy is Lactate of Zinc. In six months, from four to nine ounces were given. (See *Medico-Chirurgical Journal* of 1857, pages 384 and 385.)

I was so forcibly struck with this report at the time, that I concluded to give the remedy a trial the first opportunity offering. In the winter of 1859–60, such did occur. Having been appointed Assistant-Physician to the Western Asylum for Lunatics in Kentucky, and there being many epileptic cases in the establishment, I got the consent of the Superintendent to try the zinc treatment on some of the younger and most robust.

There were first selected five. The dose given was, at the commencement, one grain three times daily, and that before each meal. In these cases, the spasm came on once or twice a month, and continued for several days, always leaving a prostrated condition in the patient. These chosen patients were placed under treatment at different stages of the disease, or else of the attack; before, during, or immediately after recovery, and, in all, with a decided benefit. The paroxysms came on less frequently, and milder in their effects, leaving them in a stronger and more healthy condition than before—some would pass as many as three or four months without a recurrence.

We were thus induced to try it on all the patients in the asylum (epileptic of course), and they were very numerous, there being some 240 in the hospital. The result was an improvement in all, though not so marked as in those first selected for experiment. These first were all young, none had been affected over six years, and none less than three.

Then we tried the sulph. atropine, but though the result was not so successful, there was a perceptible improvement in the cases in which it was used; indeed, in some one or two cases, the effect seemed to be better.

Then, I was induced to combine the zinc and atropine, or, rather, the zinc and belladonna, in these proportions:—Zinci lactas grs. xxx., ext. belladon. grs. viij. M. et F. in pil. x. S. One before each meal. This proved more efficacious than either separately, and in no case have I ever used it without effectually controling the paroxysm in from 24 to 48 hours.

In 1861, whilst I was connected with the institution, one of the female patients, 27 or 28 years old, who had been affected from childhood and had suffered severely, whose mind was almost gone, never passing a week without a paroxysm, which frequently lasted a week, the spasms reöcurring so rapidly that it was difficult to say when they commenced and when they ended. In one of these attacks, commencing during the night, and continuing all night, increasing in frequency and force, I gave these pills of zinc and belladonna. The first, I gave myself, opening the mouth and, by means of a spoon, passing it beyond the epiglottis. The nurse was directed to repeat the dose in three hours. Before the fourth pill was given, there was a material abatement of the paroxysm.

I tried it several times that summer, upon her and upon others with like success; and again, in 1863–4 upon the same case, with the same effect.

On the 3d of May, 1863, I was called to see a pregnant negress with puerperal convulsions. I prescribed opium and assafœtida, not having the zinc, but with little success. Not able to find the zinc, I did find the belladonna, and used it in combination with assafœtida, with happy result, the second dose materially lessening the force of the attack, and the third relieving her for several hours, when they again returned, but not with the same force or frequency. I turned the fœtus and delivered a fine, healthy male child. The convulsions continued for 24 hours, but less frequent and of a milder character; then they subsided.

I have never seen the remedy used in the earlier stages of the disease, nor have I seen it persevered in more than three or four months. Whenever it has been used, to my knowledge, it has had a decidedly good effect; and I am strongly of the opinion that much good may result from its use in the earlier stages of the disease. It certainly deserves a trial, and I would be glad if some of your many subscribers would give it a trial and report success.

